# Assessing the effect of forcefield parameter sets on the accuracy of relative binding free energy calculations

**DOI:** 10.3389/fmolb.2022.972162

**Published:** 2022-09-12

**Authors:** Shan Sun, David J. Huggins

**Affiliations:** ^1^ Tri-Institutional Therapeutics Discovery Institute, New York, NY, United States; ^2^ Department of Physiology and Biophysics, Weill Cornell Medical College of Cornell University, New York, NY, United States

**Keywords:** forcefield, relative binding free energy, free energy perturbation (FEP), OpenMM, validation

## Abstract

Software for accurate prediction of protein-ligand binding affinity can be a key enabling tool for small molecule drug discovery. Free energy perturbation (FEP) is a computational technique that can be used to compute binding affinity differences between molecules in a congeneric series. It has shown promise in reliably generating accurate predictions and is now widely used in the pharmaceutical industry. However, the high computational cost and use of commercial software, together with the technical challenges to setup, run, and analyze the simulations, limits the usage of FEP. Here, we use an automated FEP workflow which uses the open-source OpenMM package. To enable effective application of FEP, we compared the performance of different water models, partial charge assignments, and AMBER protein forcefields in eight benchmark test cases previously assembled for FEP validation studies.

## Introduction

Accurate prediction of protein-ligand binding affinity can play an important role in hit-to-lead and lead optimization (Steinbrecher, 2012). It can accelerate drug discovery programs and improve the cost-efficiency when used to prioritize compounds for synthesis (Steinbrecher, 2012; Homeyer et al., 2014). Alchemical free energy calculations are a class of rigorous methods that can be used for binding affinity prediction (Christ et al., 2010; Chodera et al., 2011). They can compute both the absolute binding free energy (Boyce et al., 2009)^,^ (Mobley et al., 2007)^,^ (Irwin and Huggins, 2018) and, more commonly used in the pharmaceutical industry, the relative binding free energy (RBFE) between structurally related compounds (Christ et al., 2010; Steinbrecher and Labahn, 2010; Steinbrecher, 2012). RBFE calculations involve the transformation of one chemical species into another via an “alchemical” pathway. The alchemical transformation from the initial state to the final state is usually characterized by a non-physical coupling parameter λ. The free energy difference is calculated as the summation of alchemical transformation between fixed-λ states. Free energy perturbation (FEP) (Zwanzig, 1954; Bennett, 1976) and thermodynamic integration (TI) (Kirkwood, 1933)^,^ (Kirkwood, 1934)^,^ (Kirkwood, 1935) are both rigorous approaches that predict differences in protein-ligand binding affinities between congeneric molecules using molecular dynamics simulations. They are currently the most widely used approaches for RBFE calculations. (Abel et al., 2017a).

In particular, FEP is increasingly used in the pharmaceutical industry, typically in the lead optimization stage which involves synthesis of hundreds of close analogs with small structural modifications (Wade and Huggins, 2019). However, historically there have been numerous challenges limiting the success of FEP (Chodera et al., 2011). This includes inadequate sampling of relevant configurations, limited force field accuracy and technical hurdles to setup, run and analyze the calculations. (Mobley and Gilson, 2017). For example, when there are large structural reorganizations in the protein or ligand upon the alchemical transformation, large energy barriers can exist between different conformations. This can cause the protein or ligand to be trapped in a configuration during the simulation. (Gallicchio and Levy, 2011). The methods of Hamiltonian replica exchange and solute tempering (Liu et al., 2005) were developed to enhance sampling and address this issue. (Jiang and Roux, 2010).

Commercial software, such as Schrödinger’s FEP+ (Wang et al., 2015; Abel et al., 2017b), provides accurate force field parameters (Harder et al., 2016)^,^ (Lu et al., 2021) and an intuitive GUI for setting up and analyzing simulations. The FEP + protocol using replica exchange with solute tempering (REST) (Liu et al., 2005) and OPLS2.1 force field yielded an accurate free energy prediction with edgewise mean unsigned errors (MUEs) around 0.90 kcal/mol with respect to experiments on eight test cases (330 edges). (Wang et al., 2015). An orthogonal approach to free energy calculations called thermodynamic integration (TI) (Kirkwood, 1935) was also validated on the same dataset using AMBER (Salomon-Ferrer et al., 2013)^,^ (Cheatham et al., 2005) with a slightly larger overall edgewise MUE of 1.17 kcal/mol based on the cycle closure ddG. (Song et al., 2019). The FEP+ and AMBER TI validations both focus on edgewise MUEs: the MUE between experimental and predicted difference in binding affinity for all edges in the perturbation map. In this study we focus on the MUE of the compound binding affinities: the MUE between experimental and predicted binding affinity for all compounds. This provides a more direct comparison with experimental measurements, and we term it the MUE in binding affinity. For reference, we calculated the MUE in binding affinity for all 199 ligands from the FEP+ and TI studies: 0.77 kcal/mol and 1.01 kcal/mol respectively (Table 2).

Assessment of open-source MD packages for FEP and benchmarking of widely available force fields is of general interest to the community (Huggins, 2022). To explore applications of FEP calculations, we implemented an automated tool Alchaware, which performs FEP calculations using the open-source OpenMM code (Eastman and Pande, 2010a)^,^ (Eastman and Pande, 2010b)^,^ (Eastman et al., 2017)^,^ (Eastman and Pande, 2015). The performance and validity of a set of commonly used force field ^26^parameters were assessed with Alchaware on the eight test cases often referred as the JACS set for benchmarking free energy calculations. (Wang et al., 2015).

In this study, we validated FEP calculations with the widely used AMBER/GAFF forcefields (AMBER ff14SB (Maier et al., 2015)/GAFF2.11^28^) on the large dataset of eight test cases (330 edges). We selected water models that are available “out of the box” in the OpenMM package. The three-site water models are computationally efficient, therefore we chose the widely used three-site models SPC/E (Berendsen et al., 1987) and TIP3P (William et al., 1983). We also included a four-site model TIP4P-Ewald (Horn et al., 2004), which is optimized for PME calculations. We assessed the effect of these different water models on prediction accuracy. The AMBER ff15ipq protein force field, a second-generation force field developed using the Implicitly polarized charge model (IPolQ) for deriving implicitly polarized charges in the presence of explicit solvent, (Debiec et al., 2016), was compared with the AMBER ff14SB force field. Additionally, two partial charge models (AM1-BCC (Jakalian et al., 2002) and RESP (Christopher et al., 1993)) were evaluated in the FEP calculations. The five parameter sets tested are listed in Table 1.

**TABLE 1:** The five forcefield parameter sets tested.

Parameter Set	Protein Forcefield	Water Model	Charge Model
1	AMBER ff14SB	SPC/E	AM1-BCC
2	AMBER ff14SB	TIP3P	AM1-BCC
3	AMBER ff14SB	TIP4P-Ewald	AM1-BCC
4	AMBER ff15ipq	SPC/E	AM1-BCC
5	AMBER ff14SB	TIP3P	RESP
6	AMBER ff15ipq	TIP4P-Ewald	AM1-BCC

## Materials and methods

### Test set selection

The existing JACS benchmark set (Wang et al., 2015) of BACE, CDK2, JNK1, MCL1, P38, PTP1B, Thrombin and TYK2 was used for validation.

### Protein preparation

Protein structures were taken from the JACS benchmark set paper. (Wang et al., 2015). Protein N-termini were converted to a protonated amine and protein C-termini were converted to a charged carboxylate. For CDK2 (PDBID 1H1Q (Davies et al., 2002)), JNK1 (PDBID 2GMX (Szczepankiewicz et al., 2006)), MCL1 (PDBID 4HW3 (Friberg et al., 2013)), P38 (PDBID 3FLY (Goldstein et al., 2011)) and TYK2 (PDBID 4GIH (Liang et al., 2013)) there are no water molecules at these active sites. For BACE (PDBID 4DJW (Cumming et al., 2012)), PTP1B (PDBID 2QBS (Wilson et al., 2007)) and Thrombin (PDBID 2ZFF (Baum et al., 2009)), active site water molecules were retained. Ligands were aligned to a common core using the maximum common substructure. Input scripts and test set structure files are available on Github (https://github.com/shansun7994/Alchaware_v5.0).

### Forcefields

The GAFF 2.11 forcefield (Wang et al., 2004) was used for ligand parameters. Three water models, two protein forcefields, and two charge models were tested. AM1-BCC charges (Jakalian et al., 2002) were calculated using the Antechamber package (Wang et al., 2001). RESP charges (Christopher et al., 1993) were calculated with Jaguar (Bochevarov et al., 2013) using the DFT/B3LYP method (Lee et al., 1988)^,^ (Becke, 1993) (Becke, 1993)with a Poisson-Boltzmann solver and water as the solvent. The crystallographic binding modes of the ligands were first subjected to minimization at the 3–21G* level and then charges were fit at the 6–31G** level.

### FEP calculations

FEP calculations were performed using OpenMM 7.2 (Eastman et al., 2017) with the OpenMMTools toolkit for Hamiltonian replica exchange. All systems in all states were minimized with the OpenMM local energy minimizer. The equilibration was conducted in the NPT ensemble for 500ps at 300 K and 1 atm using a Monte Carlo Barostat. Production simulations for 5ns were performed with a Langevin integrator in the NPT ensemble with a timestep of 4.0 fs using hydrogen mass repartitioning and a hydrogen mass of 4 AMU (Hopkins et al., 2015)^,^ (Jung et al., 2021). The RBFEs were calculated using the MBAR estimator (Bennett, 1976; Shirts and Chodera, 2008) with 12 equally-spaced lambda windows. Two calculations were performed to estimate each relative binding free energy: conversion of molecule A to B in complex and conversion of molecule A to B in solvent. Solvent systems were generated with a 9.0 Å buffer between the solute and the edge of the cubic periodic box. Complex systems were generated with a 5.0 Å buffer between the solute and the edge of the cubic periodic box. Systems were neutralized and the ionic strength was set to 150 mM with Na+ and Cl-ions. Electrostatics were modelled using particle mesh Ewald method (Darden et al., 1993) and van der Waals were modelled using a nonbonded cutoff of 10.0 Å. Bonds to hydrogen were constrained, and water molecules were modeled as rigid. To avoid the numerical instabilities referred to as end point catastrophes that occur when ligands approach the fully decoupled state, OpenMMTools employs a softcore function. (Pham and Shirts, 2011). Default parameters were used for softcore_alpha (0.5), softcore_a (1), softcore_b (1), softcore_c (6), softcore_beta (0.0), softcore_d (1), softcore_e (1), and softcore_f (2). For transformations from molecule A to molecule B, hybrid molecules with dual topology were generated by identifying atoms shared between A and B that make a common core and atoms unique to A and B that appear and disappear. The sterics of A and B were first entirely coupled before switching the electrostatics. The predicted binding affinities are calculated using the Arsenic GitHub package (https://github.com/OpenFreeEnergy/arsenic). Perturbation networks are included in the Supported Information (Supplementary Figure S1).

## Results and discussion

**FIGURE 1:**
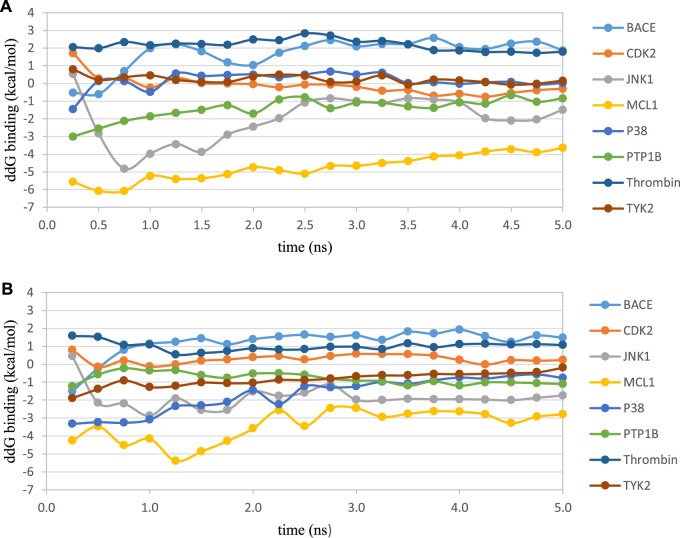
Convergence of the RBFE for the representative perturbation in each test case using the AMBER ff14SB force field with AM1-BCC charges and **(A)** SPC/E water model or **(B)** TIP3P water model. In each test case, the perturbation with the lowest similarity score (Liu et al., 2013) obtained from the Schrödinger FEP+ panel was chosen as the representative perturbation in this plot. Free energies were estimated every 0.25 ns.

**FIGURE 2:**
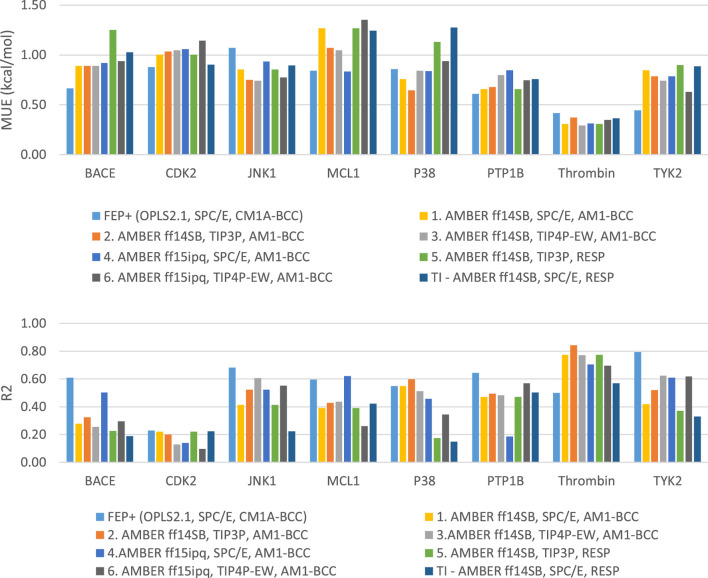
Plots of MUE and R^2^ for each target separately.

We studied the effect of the simulation parameters on Schrödinger’s JACS benchmark set, which includes eight protein targets, 199 ligands and 330 perturbations. It is worth noting that experimental uncertainties can be on the order of 0.64 kcal/mol. (Hahn et al., 2021). Starting with a parameter set using the GAFF 2.11 ligand force field, the AMBER ff14SB protein forcefield, the SPC/E water and AM1-BCC charges, the overall mean unsigned error (MUE) and root mean square error (RMSE) of 199 ligands were 0.89 kcal/mol and 1.15 kcal/mol, respectively (Table 2). With a simulation time of 5 ns per lambda window and a frequency of replica exchange at 4 ps, we examined the convergence of the representative edges in each test case (Table 3). The representative edges were chosen in each case by the lowest similarity score (Liu et al., 2013) reported in the Schrödinger FEP + panel. In general, the lower the similarity score, the higher the difficulty of the perturbation is likely to be. The total binding free energies estimated for these representative edges in each set are shown in Figure 1A. In all cases, predictions show reasonably good convergence after 2.5 ns. A repeat run of these perturbations was carried out using a different initial configuration by minimizing the protein structures. The ddG difference between the two configurations is within 1.0 kcal/mol for all test cases except BACE where the ddG difference between the two configurations is within 2.0 kcal/mol (Supplementary Figure S2). The overall accuracy of the prediction (MUE 0.89 kcal/mol, RMSE 1.15 kcal/mol) is better than the validation reported using TI (MUE 1.01 kcal/mol, RMSE 1.3 kcal/mol), and comparable to the commercial software FEP + using the OPLS2.1 force field and SPC/E water model (MUE 0.77 kcal/mol, RMSE 0.93 kcal/mol), though errors are a little larger (Table 2). Notably better performance was seen in the case of JNK1 with an improvement of 0.22 kcal/mol in MUE (Figure 2; Table 4). However, concerns were raised with the quality of the JNK1 structure (2GMX) used for benchmarking. (Hahn et al., 2021). The high R-free value (0.351), as well as the large difference between R-value and R-free for this JNK1 structure, indicates a possible overfit of the atomic model to the experimental diffraction pattern when solving the crystal structure. The coordinate error which assesses the precision of the model is 0.77. This does not fulfill the high-quality structure criteria (<0.7). (Hahn et al., 2021).

**TABLE 2:** Summary of accuracy and correlation statistic results of the five parameter sets tested here alongside two published datasets.

	FEP+ ^18^	AMBER TI ^22^	Alchaware					
	OPLS2.1	AMBER ff14SB	1. AMBER ff14SB	2. AMBER ff14SB	3. AMBER ff14SB	4. AMBER ff15ipq	5. AMBER ff14SB	6. AMBER ff15ipq
	SPC/E	SPC/E	SPC/E	TIP3P	TIP4P-EW	SPC/E	TIP3P	TIP4P-EW
	CM1A-BCC	RESP	AM1-BCC	AM1-BCC	AM1-BCC	AM1-BCC	RESP	AM1-BCC
MUE (kcal/mol)	0.77	1.01	0.89	0.82	0.85	0.85	1.03	0.95
RMSE (kcal/mol)	0.93	1.3	1.15	1.06	1.11	1.07	1.32	1.23
^a^R^2^	0.66	0.44	0.53	0.57	0.56	0.58	0.45	0.49
^a^ρ	0.82	0.65	0.7	0.75	0.73	0.74	0.65	0.70
^a^τ	0.62	0.48	0.52	0.56	0.54	0.55	0.47	0.51

^a^Correlation coefficient (R^2^), Spearman’s rank (ρ), and Kendall rank correlation coefficient (τ) of 199 compounds

**TABLE 3:** The representative perturbations used to explore convergence for the eight test cases.

Test Case	Ligand 1	Ligand 2	Similarity score
BACE	CAT-13g	CAT-17i	0.33
	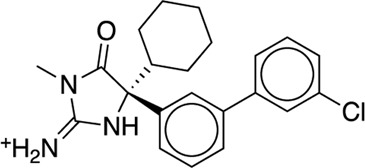	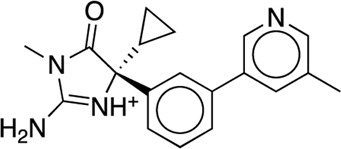	
CDK2	30	31	0.09
	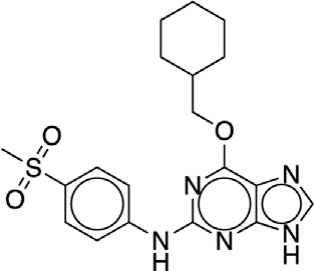	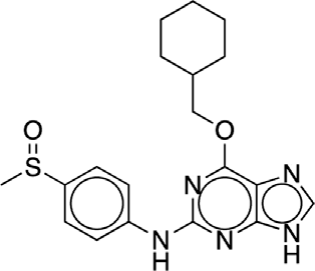	
JNK1	18626-1	18660-1	0.41
	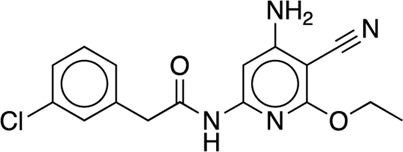	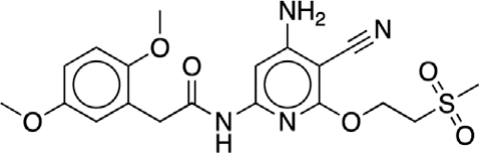	
MCL1	29	40	0.33
	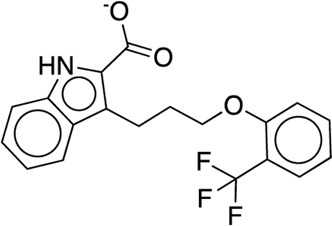	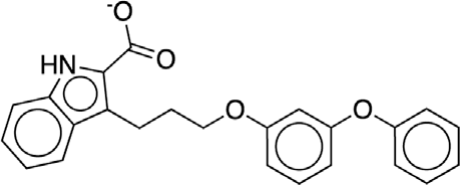	
P38	p38a_2g	p38a_2c	0.22
	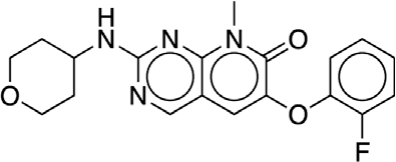	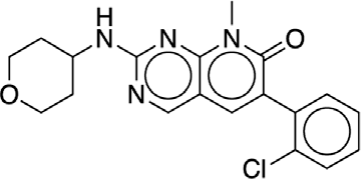	
PTP1B	23469	20669	0.18
	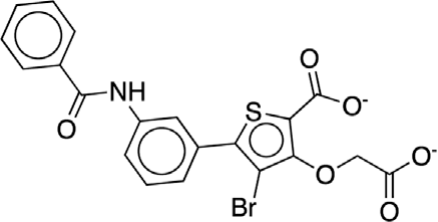	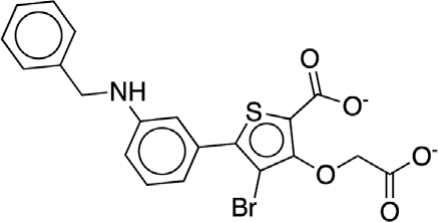	
Thrombin	1a	3b	0.74
	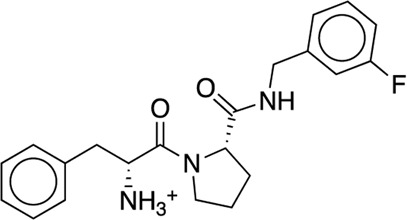	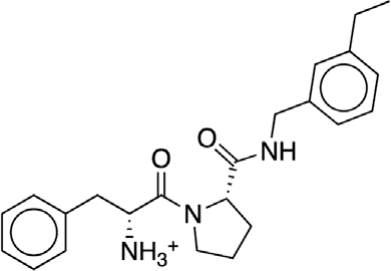	
TYK2	Ejm_49	Ejm_50	0.45
	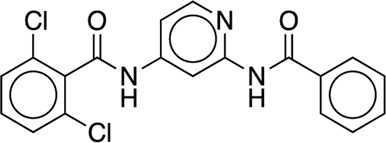	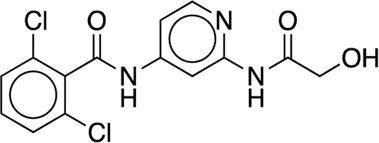	

**TABLE 4:** Summary of MUE, RMSE and R^2^ of 8 test cases and parameters.

Target		BACE	CDK2	JNK1	MCL1	P38	PTP1B	Thrombin	TYK2
FEP+	MUE	0.67	0.88	1.07	0.84	0.86	0.61	0.42	0.45
	RMSE	0.85	1.04	1.15	1.04	0.99	0.80	0.54	0.57
	R^2^	0.61	0.23	0.68	0.60	0.55	0.64	0.50	0.79
1. AMBER ff14SB, SPC/E, AM1-BCC	MUE	0.89	1.00	0.85	1.27	0.76	0.66	0.31	0.85
	RMSE	1.15	1.24	0.96	1.53	0.94	1.05	0.40	1.04
	R^2^	0.28	0.22	0.41	0.39	0.55	0.47	0.77	0.42
2. AMBER ff14SB, TIP3P, AM1-BCC	MUE	0.89	1.03	0.75	1.07	0.65	0.68	0.37	0.78
	RMSE	1.12	1.36	0.87	1.36	0.77	0.94	0.47	0.91
	R^2^	0.32	0.20	0.52	0.43	0.60	0.49	0.84	0.52
3. AMBER ff14SB, TIP4P-EW, AM1-BCC	MUE	0.89	1.05	0.74	1.05	0.84	0.80	0.29	0.74
	RMSE	1.15	1.38	0.90	1.32	1.11	0.98	0.36	0.91
	R^2^	0.25	0.13	0.61	0.44	0.51	0.48	0.77	0.62
4. AMBER ff15ipq, SPC/E, AM1-BCC	MUE	0.92	1.06	0.94	0.83	0.84	0.85	0.31	0.78
	RMSE	1.09	1.37	1.04	1.02	1.02	1.22	0.39	0.98
	R^2^	0.50	0.14	0.52	0.62	0.46	0.19	0.70	0.61
5. AMBER ff14SB, TIP3P, RESP	MUE	1.25	1.00	0.85	1.27	1.13	0.66	0.31	0.90
	RMSE	1.57	1.24	0.96	1.53	1.47	1.05	0.40	1.06
	R^2^	0.22	0.22	0.41	0.39	0.17	0.47	0.77	0.37
6. AMBER ff15ipq, TIP4P-EW, AM1-BCC	MUE	0.94	1.14	0.78	1.35	0.94	0.75	0.35	0.63
	RMSE	1.22	1.52	0.95	1.64	1.18	0.93	0.41	0.80
	R^2^	0.30	0.10	0.55	0.26	0.34	0.57	0.70	0.62
TI - AMBER ff14SB, SPC/E, RESP	MUE	1.03	0.90	0.90	1.24	1.28	0.76	0.37	0.89
	RMSE	1.32	1.08	1.13	1.48	1.62	1.01	0.51	1.13
	R^2^	0.19	0.22	0.22	0.42	0.15	0.50	0.57	0.33

We then used the same ligand and protein force fields to test whether the three-point TIP3P and four-point TIP4P-Ewald water models would improve the prediction accuracy. Both the TIP3P and TIP4P-Ewald water models slightly improve the overall performance with lower error and higher correlation coefficient compared to SPC/E water model (Table 2, parameter set 1, 2 and 3). This better performance could be due to the improvement of the convergence (Figure 1).

Using the GAFF 2.11 ligand force field, SPC/E water model and AM1-BCC charge, we found the AMBER ff15ipq force field (Table 2, parameter set 4), which better models the polarization effect, has a small improvement in the overall accuracy of ddG predictions (MUE 0.85 kcal/mol, RMSE 1.07 kcal/mol). This improvement is more notable in the MCL1 case, where the carboxylic acid group of all the ligands forms a critical salt bridge with the ARG263 residue. In this case, the AMBER ff15ipq force field improved the MUE by 0.44 kcal/mol compared to AMBER ff14SB (Figure 2, parameter set 1, 4). However, the four-point TIP4P-EW water model does not improve the accuracy on AMBER ff15ipq (Table 2, parameter set 6). A recent benchmark by Huai et al. evaluated AMBER protein force fields using a small test set has found that AMBER ff14SB and AMBER ff19SB (Tian et al., 2020) both perform well in alchemical calculation (Huai et al., 2021). There are problems with using the AMBER ff19SB force field in OpenMM due to the use of CMAP terms, but it should be explored in future work. In addition, the widely used CHARMM36 (Huang and MacKerell, 2013) and CHARMM36m (Huang et al., 2017) protein forcefield are also worth exploring.

The restrained electrostatic potential (RESP) approach is a commonly-used method of assigning partial charges to organic compounds (Christopher et al., 1993; Wang et al., 2000). Surprisingly, using RESP charges instead of the AM1-BCC charge does not tend to improve prediction accuracy or correlation (MUE 1.03 kcal/mol, RMSE 1.32 kcal/mol, *R* ^2^ 0.45). Song et al. (Song et al., 2019) validated the performance of similar force field parameters (AMBER ff14SB/GAFF1.8 force field, SPC/E, and RESP charges) using thermodynamic integration (TI) free energy calculation approach on the same benchmark set gave similar results (MUE 1.01 kcal/mol, RMSE 1.30 kcal/mol, *R* ^2^ 0.44) (Table 2, TI and parameter set 5). This suggested the differences in performance arise largely from the charge model employed.

**FIGURE 3:**
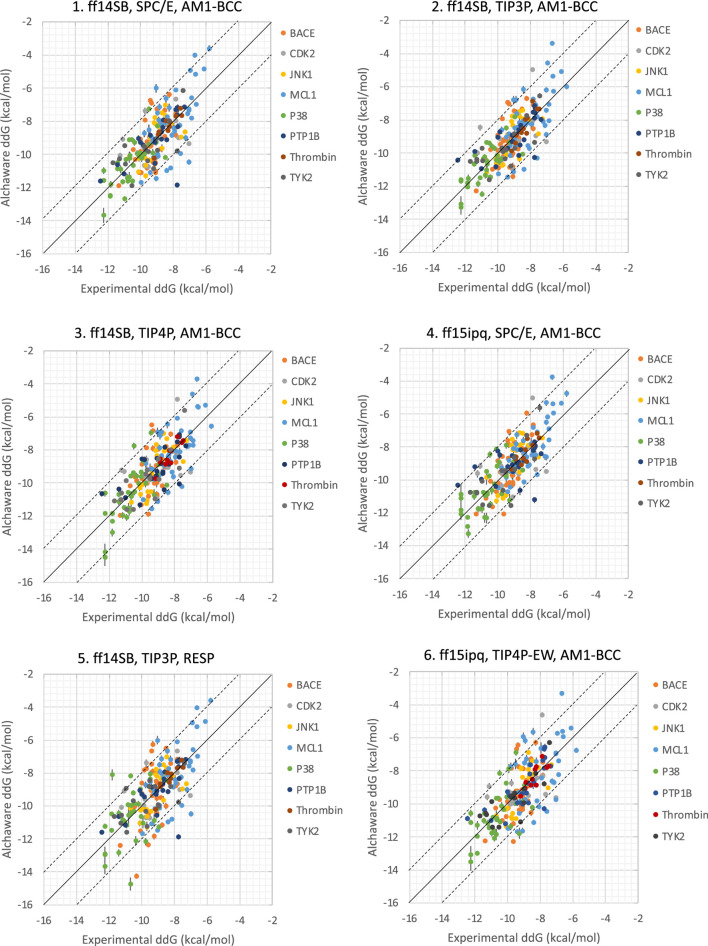
Correlation between predicted binding free energies and experimental data with 6 parameter sets. Error bars indicate the cycle closure error.

Together, the parameter set 2 (AMBER ff14SB, TIP3P, AM1-BCC) has the best performance in the JACS benchmark set (Figure 3). For the 199 ligands, the majority of the binding free energy values are within 2.0 kcal/mol except for 17 ligands. Notably, these ligands are for BACE (3 ligands), MCL1 (9 ligands) and PTP1B (2 ligands) where the ligands are charged. The accuracy and correlation between the parameter sets are generally aligned, such that a high accuracy model also does better in ranking compounds. In cases where salt-bridges are formed between protein and ligand, the AMBER ff15ipq protein force field tends to increase the prediction accuracy.

## Conclusion

We developed a workflow for calculating FEP RBFEs with an automated tool Alchaware using OpenMM. Validations of the FEP calculations with open-source force field parameters were carried out on the JACS benchmark set of eight test cases.

TIP3P and TIP4P-Ewald water models slightly improved the overall performance relative to SPC/E, with lower error and higher correlation coefficient with AMBER ff14SB protein force field and AM1-BCC charge. The AMBER ff15ipq protein force field (which was built to better model polarization effects) also improves the accuracy and correlation, particularly in cases where charged ligands form salt bridge interactions. This is particularly true in the case of MCL1, where the ligand forms a critical salt bridge with the charged residue (ARG263). Unfortunately, there is no improvement when using RESP charges relative to AM1-BCC charges. However, alternative protocols to generate the RESP charges should be explored in future work.

In summary, this work reports the predictive accuracy with 6 parameter sets in calculating RBFEs using FEP. Among those, set 2 (AMBER ff14SB/GAFF2.1 force field, TIP3P water model, and AM1-BCC changes) yields the best accuracy in 199 ligands (overall MUE 0.82 kcal/mol, RMSE 1.06 kcal/mol). Although the overall accuracy is not quite as good as the commercial FEP + results, in some cases (such as P38, PTP1B, and Thrombin) the accuracy is comparable. Although the better performance was seen in the case of JNK1, the protein structure used (2GMX) does not fulfill the high-quality structure criteria. This issue flags the importance of adopting best practices in constructing, preparing, and evaluating FEP calculations (Mey et al., 2020; Hahn et al., 2021). Finally, most of the poorly predicted compounds (MUE >2.0 kcal/mol) fall into three cases (BACE, MCL1 and PTP1B), where the ligands are charged. This suggests that better accuracy may be achieved by better models of charge and/or polarization and future work should be focused in this area.

## Data Availability

The original contributions presented in the study are included in the article/Supplementary Material, further inquiries can be directed to the corresponding author.
