# Supplementary material for: Assessing the effect of forcefield parameter sets on the accuracy of relative binding free energy calculations
Source: Front Mol Biosci. 2022 Sep 12;9:972162. doi: 10.3389/fmolb.2022.972162 (PMC9549959; doi:10.3389/fmolb.2022.972162)
Supplement: Supplementary file 1 [file DataSheet1.docx]

**Supporting Information**

Assessing the effect of forcefield parameter sets on the accuracy of relative binding free energy calculations

Shan Sun ^a^, David J. Huggins ^a,b^

^a^Tri-Institutional Therapeutics Discovery Institute, New York, NY 10021, USA

^b^Department of Physiology and Biophysics, Weill Cornell Medical College of Cornell University, New York, NY 10065, USA

Corresponding Author: David J. Huggins

Email: [dhuggins@tritdi.org](mailto:dhuggins@tritdi.org)

Updated: Aug 08 2022

| BACE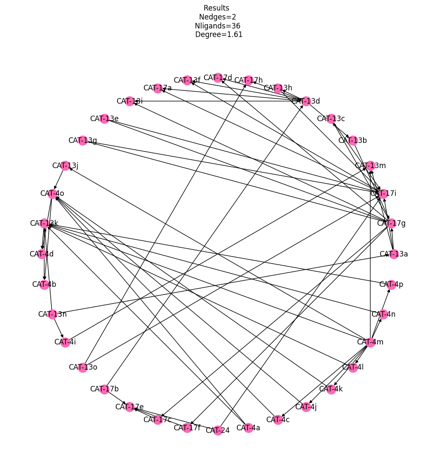 | CDK2  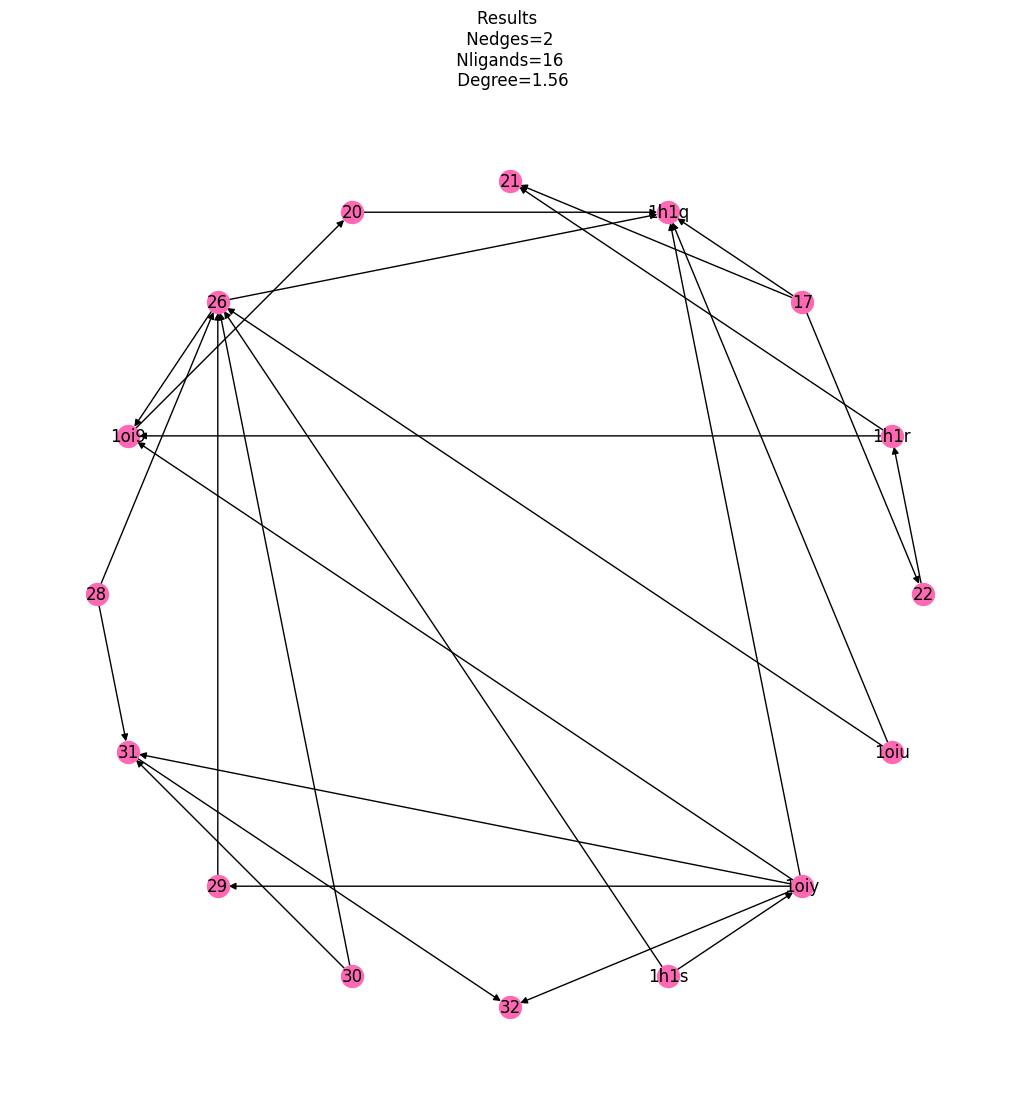 | JNK1  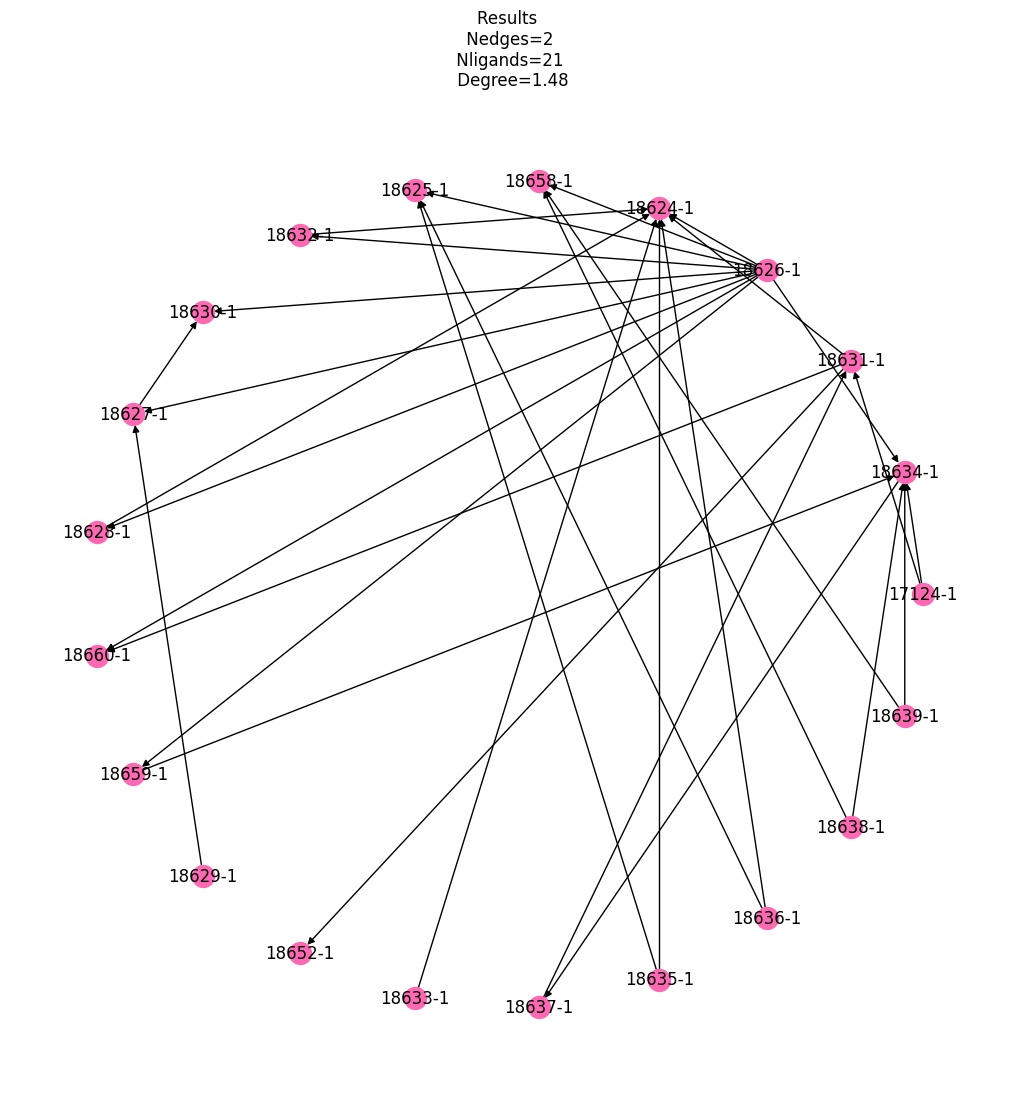 |
| --- | --- | --- |
| MCL1 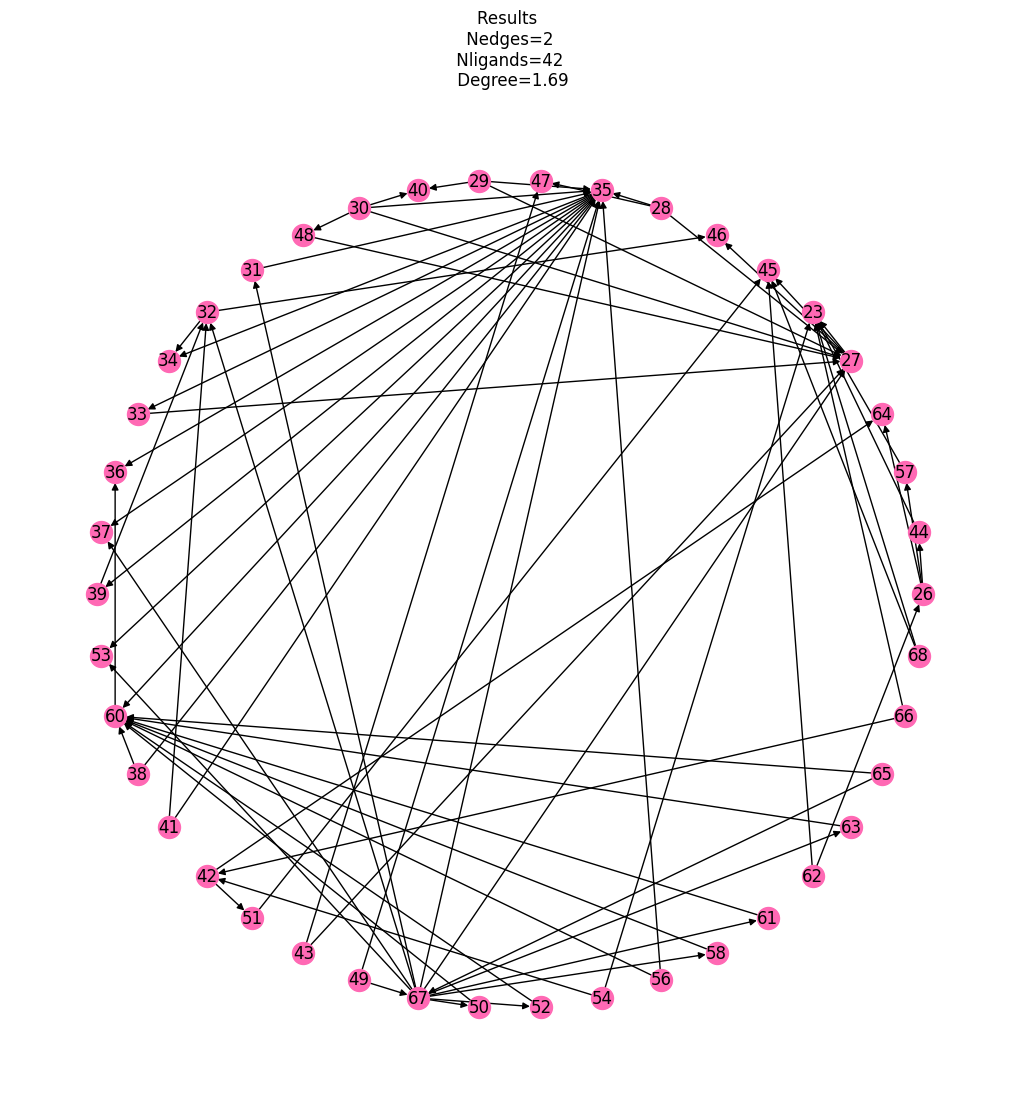 | P38  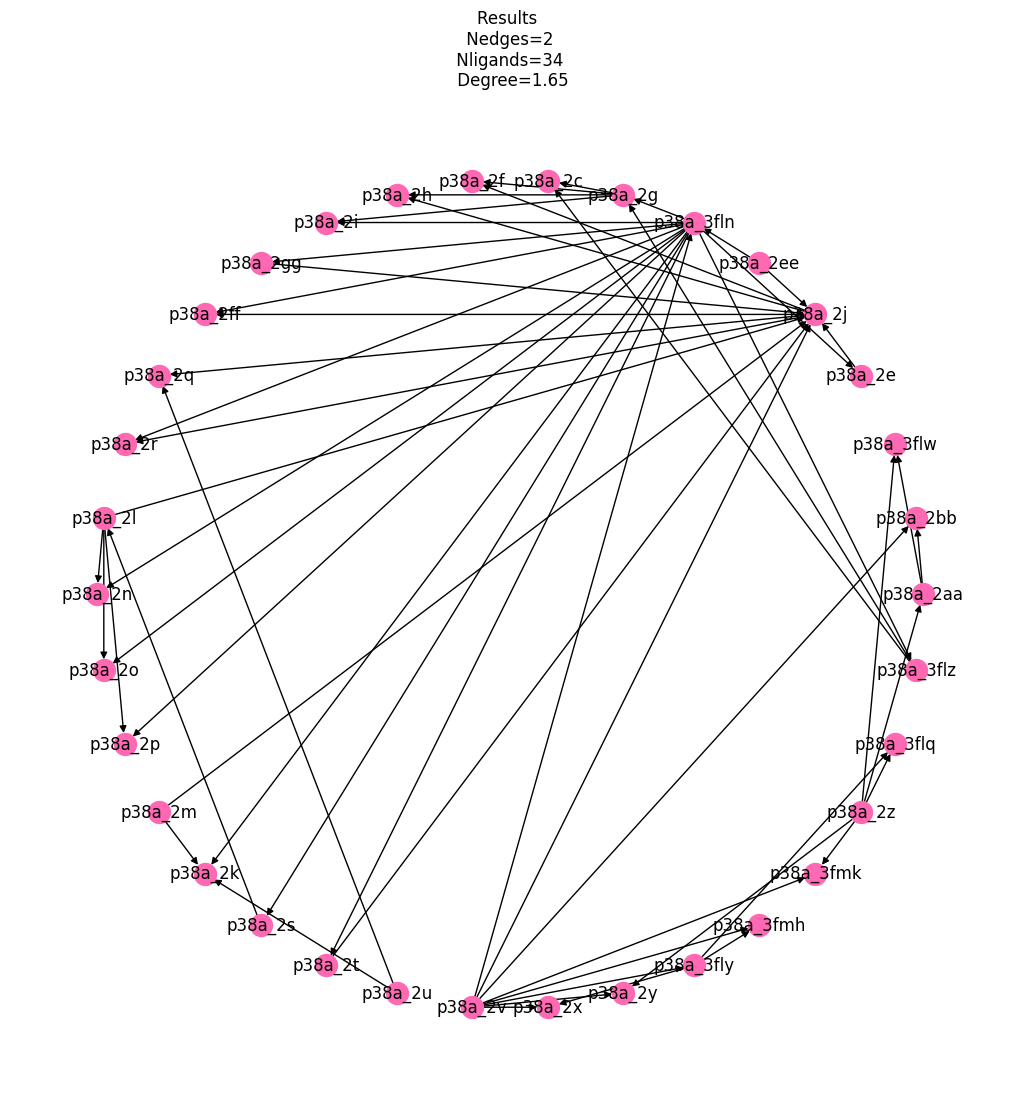 | PTP1B  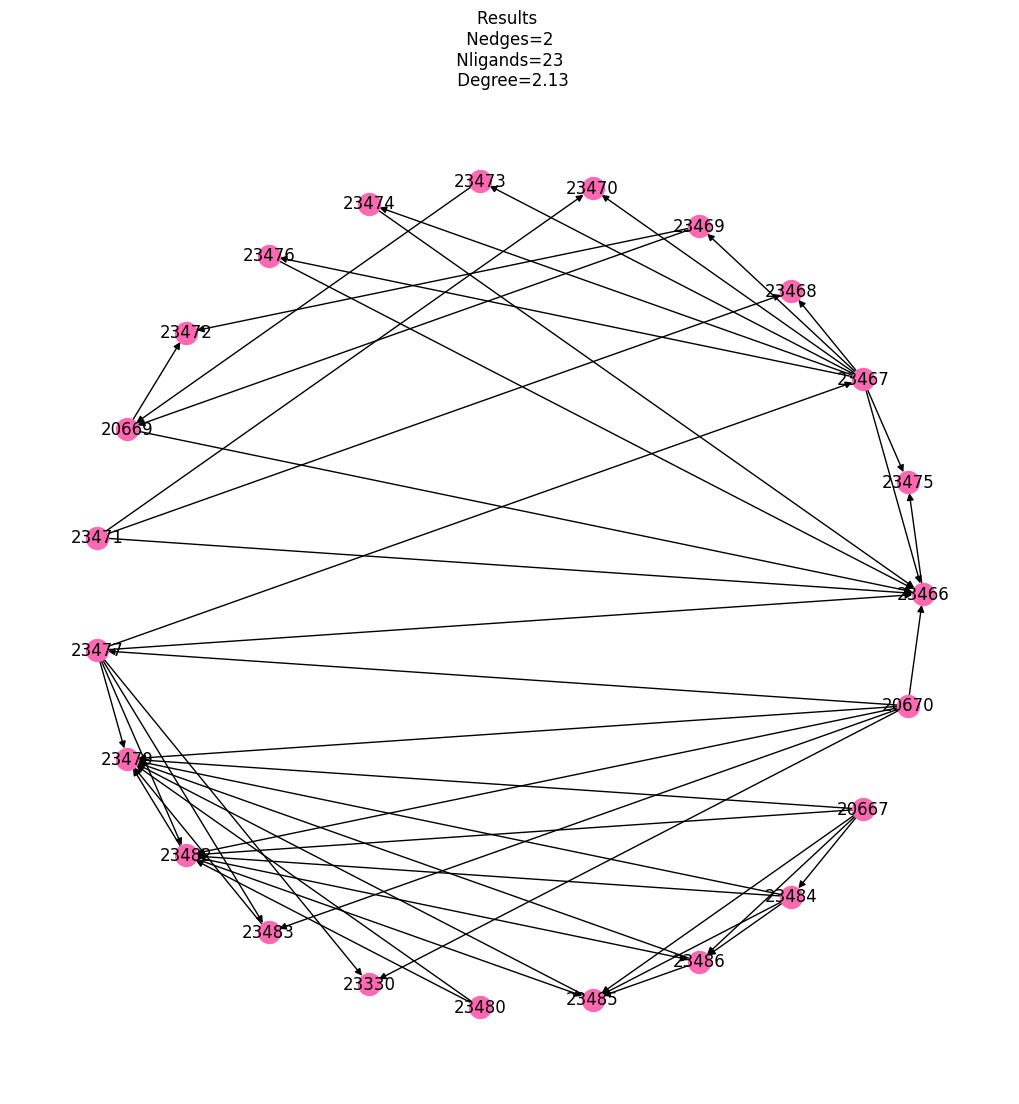 |
| Thrombin  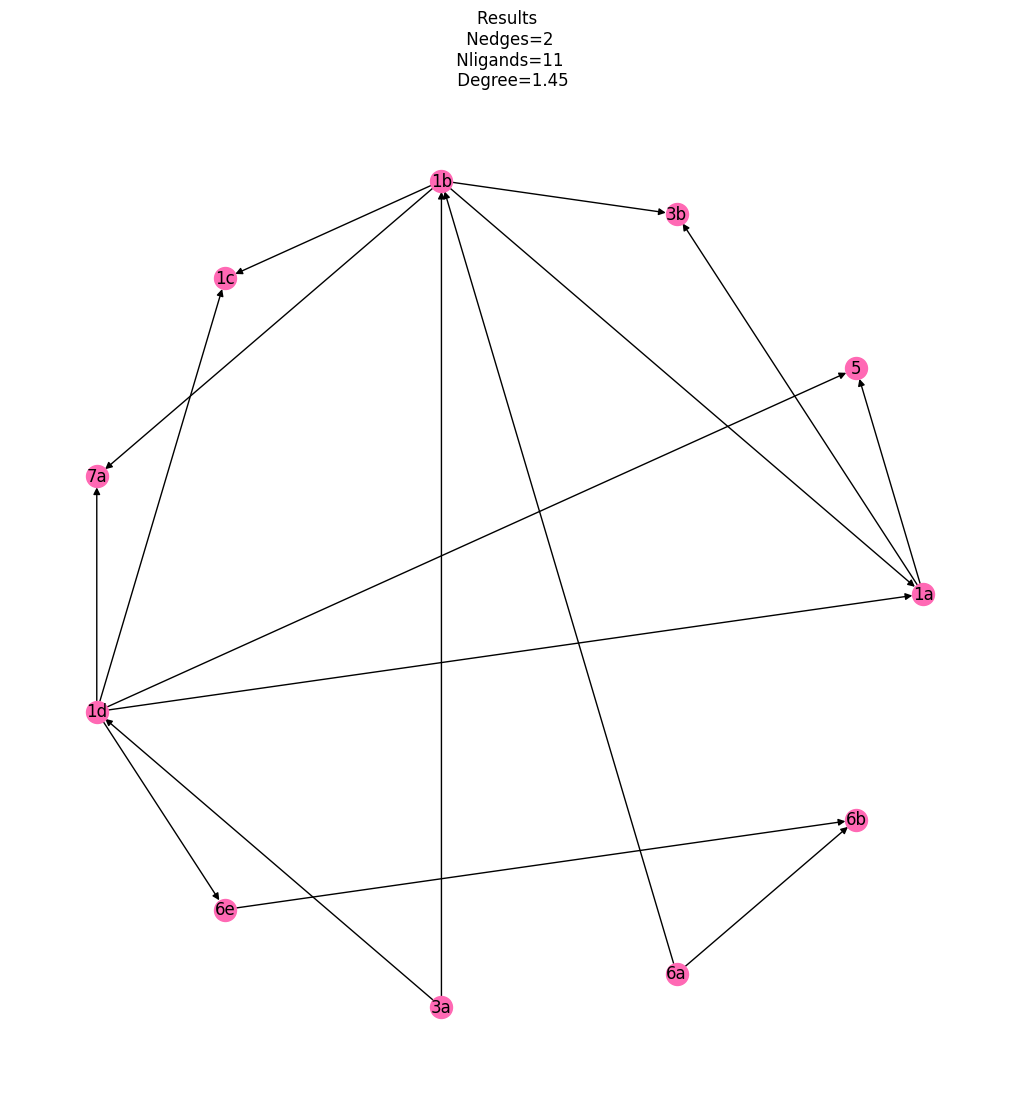 | TYK2  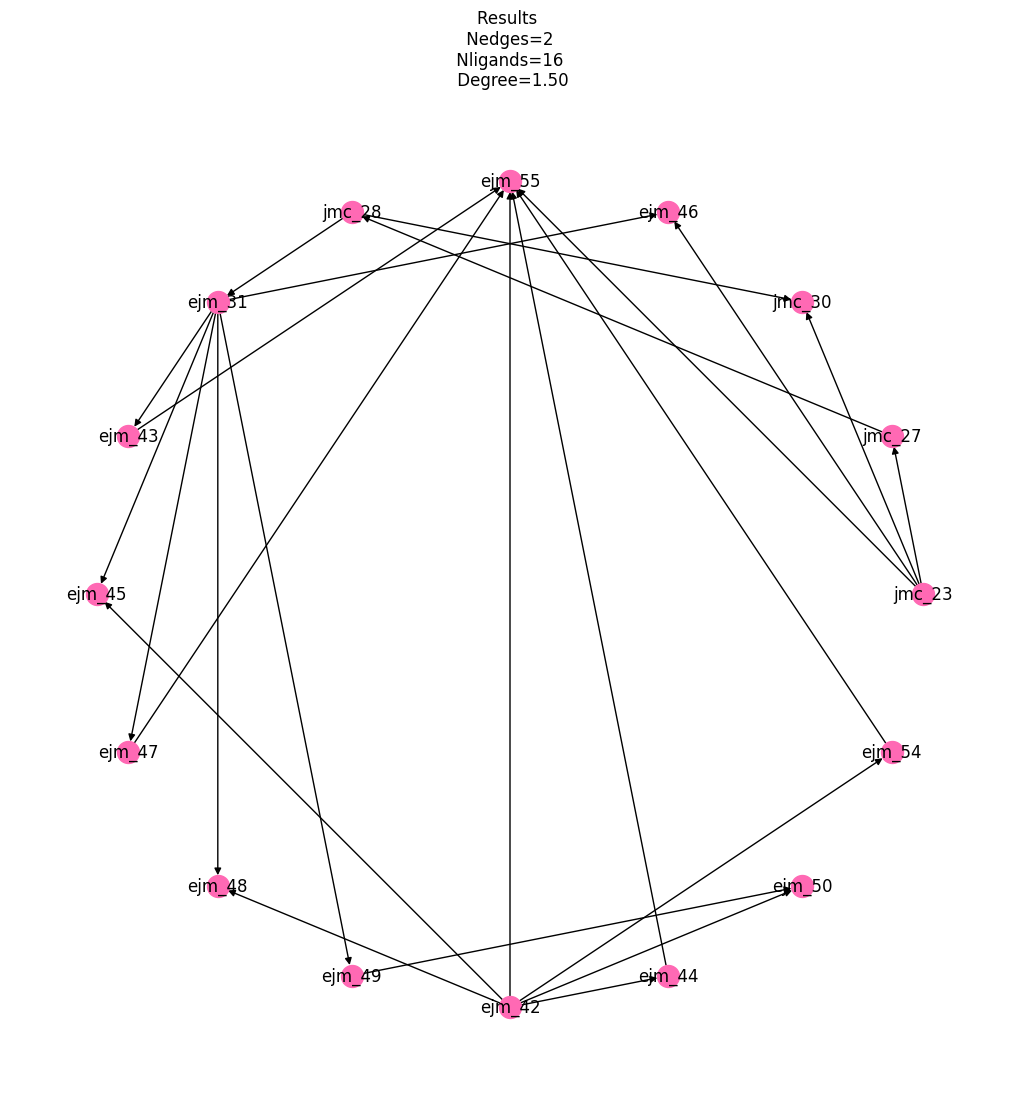 |  |

Figure S1. The Perturbation network for eight test cases.^1^

Figure S2. Results for representative perturbations (Table 3) with different initial configurations using parameter set 2.

Figure S3. Repeats of representative perturbations (Table 3) using parameter set 2.
